# *IRS1* DNA promoter methylation and expression in human adipose tissue are related to fat distribution and metabolic traits

**DOI:** 10.1038/s41598-017-12393-5

**Published:** 2017-09-28

**Authors:** Kerstin Rohde, Matthias Klös, Lydia Hopp, Xuanshi Liu, Maria Keller, Michael Stumvoll, Arne Dietrich, Michael R. Schön, Daniel Gärtner, Tobias Lohmann, Miriam Dreßler, Peter Kovacs, Hans Binder, Matthias Blüher, Yvonne Böttcher

**Affiliations:** 1 IFB Adiposity Diseases, University of Leipzig, Leipzig, Germany; 2 Department of Clinical and Molecular Biology and Akershus University Hospital, University of Oslo, Lørenskog, Norway; 3 Interdisciplinary Centre for Bioinformatics, University of Leipzig, Leipzig, Germany; 4 Department of Computer Science, University of Leipzig, Leipzig, Germany; 5 Department of Clinical Sciences, Skåne University Hospital, Malmö, Sweden; 6 Department of Medicine, University of Leipzig, Leipzig, Germany; 7 Department of Surgery, University of Leipzig, Leipzig, Germany; 8 Clinic of Visceral Surgery, Städtisches Klinikum Karlsruhe, Karlsruhe, Germany; 9 Municipal Clinic Dresden-Neustadt, Dresden, Germany; 10 University of Oslo, Institute of Clinical Medicine, Department of Clinical and Molecular Biology; Akershus University Hospital, 1478 Lørenskog, Norway

## Abstract

The SNP variant rs2943650 near *IRS1* gene locus was previously associated with decreased body fat and *IRS1* gene expression as well as an adverse metabolic profile in humans. Here, we hypothesize that these effects may be mediated by an interplay with epigenetic alterations. We measured *IRS1* promoter DNA methylation and mRNA expression in paired human subcutaneous and omental visceral adipose tissue samples (SAT and OVAT) from 146 and 41 individuals, respectively. Genotyping of rs2943650 was performed in all individuals (*N* = 146). We observed a significantly higher *IRS1* promoter DNA methylation in OVAT compared to SAT (N = 146, *P* = 8.0 × 10^−6^), while expression levels show the opposite effect direction (N = 41, *P* = 0.011). OVAT and SAT methylation correlated negatively with *IRS1* gene expression in obese subjects (N = 16, *P* = 0.007 and *P* = 0.010). The major T-allele is related to increased DNA methylation in OVAT (N = 146, *P* = 0.019). Finally, DNA methylation and gene expression in OVAT correlated with anthropometric traits (waist- circumference waist-to-hip ratio) and parameters of glucose metabolism in obese individuals. Our data suggest that the association between rs2943650 near the *IRS1* gene locus with clinically relevant variables may at least be modulated by changes in DNA methylation that translates into altered *IRS1* gene expression.

## Introduction

Body fat composition is a more precise parameter than body mass index (BMI) to analyze fat distribution. To identify genetic factors influencing body fat mass and body fat distribution large, genome wide association studies (GWAS) have been conducted^1–3^ Genes in close vicinity of the identified genetic variants and conferring important pathophysiological functions in metabolism are plausible candidate genes for further analysis on the link between adverse fat distribution, metabolic and cardiovascular diseases^4^. One such example is the previously reported single nucleotide polymorphism (SNP) rs2943650 near the *insulin receptor substrate 1* (*IRS1*) gene locus. T-allele carriers showed decreased body fat percentage along with an unfavorable metabolic profile including decreased high density lipoprotein- (HDL) cholesterol, increased triglyceride concentrations and insulin resistance^1^. IRS1 is an important player in insulin signaling being one of the most relevant proteins able to bind the phosphorylated insulin receptor to activate downstream cascades^5,6^. *IRS1* knock out mouse models further suggests that IRS1 plays a role in adipocyte differentiation^7^. Interestingly, the SNP variant rs2943650 was further shown to be associated with reduced subcutaneous adipose tissue in men^1^. Kilpeläinen *et al*. hypothesized that the SNP may lead to a predominant deposition of fat into visceral depots thereby contributing to insulin resistance and dyslipidemia, despite an overall decrease in body fat percentage. Furthermore, the body fat decreasing T-allele was shown the be associated with reduced *IRS1* mRNA expression in human subcutaneous and omental adipose tissue (SAT and OVAT)^1,8^.

However, it is still an open question by which mechanisms rs2943650 contributes to regulation of IRS1 expression and subsequent alterations in metabolic variables.

Changes in DNA methylation pattern were described as highly dynamic in response to weight loss after bariatric surgery^9^, exercise^10,11^ and between different adipose tissue compartments^12^. Previously, we identified differences in DNA methylation between SAT and OVAT^13,14^. It has been demonstrated that genetic variants influence the DNA methylation of nearby CpG sites by introducing or deleting CpG sites (CpG-SNPs)^15–17^. Several CpG-SNPs, which partially change the methylation at the corresponding and/or surrounding CpG sites associate with alternative splicing events in human pancreatic islets^16^. A recent study identified a haplotype within *TOMM20* which is related to changes in the methylation state of a core CpG site matching a relevant transcription factor binding site and to altered LDL-cholesterol levels in patients with metabolic syndrome^18^. These and several further studies provide evidence for triangular relationships between genetic variants, DNA methylation and gene expression and/or diseases state^19–21^. Hence, we hypothesized a potential interplay between the genetic variant rs2943650 near *IRS1* and epigenetic variation, such as DNA methylation within its promoter region.

To test whether the observed effects of rs2943650 near *IRS1* on expression and metabolic profiles can be mediated by an interplay with DNA methylation, we measured i) differential *IRS1* DNA methylation and mRNA expression in paired samples of human SAT vs. OVAT. We tested for ii) correlation of DNA methylation with *IRS1* gene expression levels and analyzed for association of rs2943650 effect allele (T) with both methylation and expression. iii) Finally, we performed correlation analysis to assess potential relationships between DNA methylation, mRNA expression and rs2943650 genotypes with metabolic and anthropometric variables.

## Methods

### Study population

A total of 146 individuals (men *N* = 55; women *N* = 91) from a Caucasian cohort (Germany) were included for DNA methylation analysis and SNP genotyping. Main characteristics of the study cohort are reported in Table 1. Paired samples of SAT and OVAT were obtained during open abdominal surgery for e.g. sleeve gastrectomy, Roux-en-Y gastric bypass, gastric banding, cholecystectomy, abdominal injuries or explorative laparotomy. Individuals with body weight fluctuations (partially self-reported) in the last 3 months before surgery were excluded (>2% fluctuations of body weight). mRNA expression data from either SAT or OVAT was available for 63 individuals^14^. Paired expression data from OVAT and SAT was available from 41 individuals and was used for phenotype-association analysis per adipose tissue depot. For subsequent analyses comparing both tissue depots we used 28 of these subjects for which overlapping paired methylation and gene expression data from SAT and OVAT were available. All analyses were performed in obesity-subgroups (lean, overweight and obese) if not described otherwise. Phenotyping of the study participants was performed as previously described^22^ and included anthropometric measurements (body weight, height, waist-to-hip-ratio (WHR)), body fat mass using bioimpedance analysis or dual-energy X-ray absorptiometry. Furthermore, metabolic parameters such as fasting plasma glucose (FPG) and insulin (FPI), a 75 g oral glucose tolerance test (OGTT), glycated haemoglobin (HbA1c), lipoprotein-, triglyceride-, free fatty acid- and cytokine- serum concentrations were obtained. Insulin sensitivity was assessed with hyperinsulinemic-euglycemic clamps. Determination of visceral- and subcutaneous fat areas was performed based on computed tomography or MRI scans. By setting a ratio of visceral/subcutaneous fat area of 0.5 obese individuals were further divided being either viscerally or subcutaneously obese (<0.5 = subcutaneous obesity; >0.5 = visceral obesity; 0.5 = obese (not specified)). The study was approved by the Ethics Committee of the University of Leipzig (approval no: 159-12-21052012), and performed in accordance to the declaration of Helsinki. All subjects gave written informed consent before taking part in this study.

**Table 1:** Characteristics of the study cohort.

	Total	Lean	Overweight	Obese	*P*-valuelean vs obese
N	146	50	28	68	50/68
Sex (m/f)	55/91	20/30	14/14	21/47	—
T2D (yes/no)	42/104	7/43	7/21	28/40	—
Age (years)	58 ± 16	66 ± 12	67 ± 12	49 ± 15	n.s.
Weight (kg)	100 ± 43	63 ± 10	78 ± 11	137 ± 36	<0.001
Height (m)	1.68 ± 0.90	1.67 ± 0.8	1.70 ± 0.12	1.68 ± 0.81	n.s.
BMI (kg/m^2^)	35.3 ± 14.7	22.4 ± 2.5	26.9 ± 1.4	48.3 ± 11.7	<0.001
Visceral fat area (cm²)	178 ± 160	54 ± 41.5	134 ± 76	296 ± 161	<0.001
Subcutanoues fat area (cm²)	720 ± 775	55.5 ± 27.5	271 ± 173	1438 ± 601	<0.001
CT-ratio (OVAT/SAT fat area)	0.66 ± 0.89	1.21 ± 1.25	0.63 ± 0.40	0.22 ± 0.11	<0.001
Waist circumference (cm)	106 ± 32	79 ± 17	94.5 ± 15	134 ± 21	0.022
Hip circumference (cm)	113 ± 30	87.5 ± 11	100 ± 12	140 ± 24	<0.001
Waist-to-hip ratio	0.94 ± 0.12	0.89 ± 0.11	0.95 ± 0.12	0.96 ± 0.13	n.s.
Body fat %	30.9 ± 12.8	20.8 ± 4.7	25.9 ± 4.6	43.3 ± 10.2	<0.001
Fasting plasma glucose (mmol/l)	6.03 ± 1.76	5.57 ± 1.00	5.95 ± 1.33	6.40 ± 2.23	0.011
2 hr OGTT plasma glucose (mmol/l)	6.91 ± 2.76	6.18 ± 1.49	6.2 ± 1.02	7.85 ± 3.69	n.s.
Fasting plasma Insulin (mmol/l)	73.12 ± 120.87	11.77 ± 22.18	49.30 ± 57.68	138.83 ± 157.71	<0.001
HbA1c %	5.80 ± 0.78	5.43 ± 0.54	5.79 ± 0.67	6.10 ± 0.86	<0.001
HDL cholesterol (mmol/l)	1.36 ± 0.43	1.48 ± 0.49	1.39 ± 0.33	1.19 ± 0.37	n.s.
LDL cholesterol (mmol/l)	3.20 ± 1.09	3.08 ± 1.13	3.42 ± 0.93	3.21 ± 1.13	n.s.
Triglycerides (mmol/l)	1.39 ± 0.74	1.29 ± 0.67	1.21 ± 0.43	1.57 ± 0.88	n.s.
Adiponectin (ug/ml)	9.32 ± 5.74	13.74 ± 6.78	8.94 ± 3.86	6.59 ± 3.36	<0.001

N = Number of subjects; T2D = diagnosed Type 2 Diabetes; BMI = Body mass index (WHO classification: lean ≥ 18; <25 kg/m²; <30 kg/m²obese ≥ 30 kg/m²); SAT = subcutaneous adipose tissue; OVAT = omental visceral adipose tissue; OGTT = oral glucose tolerance test; HDL = high density lipoprotein cholesterol; LDL = low density lipoprotein cholesterol. *P*-values were generated using unpaired t-tests.

### DNA extraction and bisulfite conversion

DNA extraction and bisulfite conversion was carried out in the entire cohort (N = 146). After collecting adipose tissue, samples were frozen in liquid nitrogen and stored at −80 °C. Genomic DNA was extracted using GenElute^TM^ Mammalian Genomic DNA Miniprep Kit (SIGMA-ALDRICH, USA) and were bisulfite converted using Qiagen EpiTect Fast DNA Bisulfite Kit (Qiagen, Hilden, Germany) according to the manufacturer’s protocols.

### Analysing CpG methylation within *IRS1* promoter region

**Figure 1:**
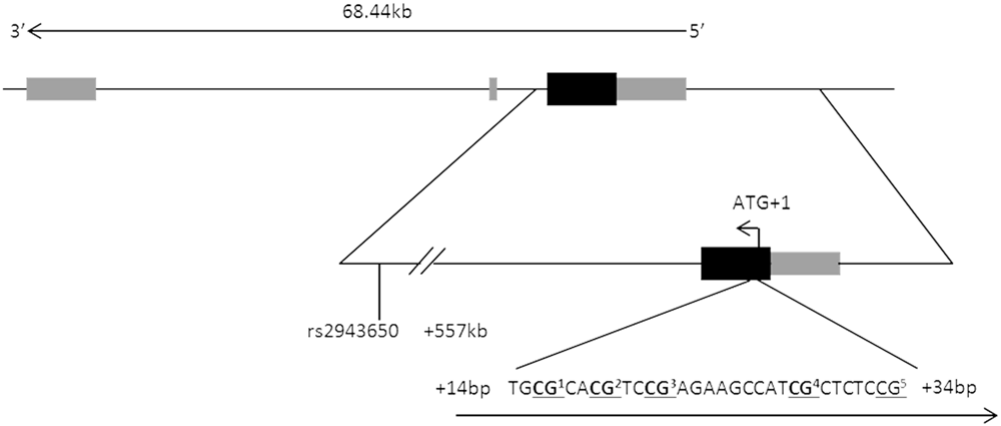
Schematic representation of *IRS1* gene locus, analyzed CpG locus and SNP location. *IRS1* exons are shown as filled boxes (black = coding exons; grey = non-coding exons). The CpG locus and analyzed SNP variant rs2943650 (C/T) are shown relative to the translation start site (ATG + 1). Analyzed CpGs are highlighted in bold and numbered according to order of analysis (CpG^5^ was excluded from analysisdue to optimization of dispensation order in the PyroMark run). Figure not scaled. kb = kilobases; bp = base pairs.

To analyse DNA methylation at CpG sites we used the Hs_IRS1_05_PM PyroMark CpG assay (PM00011711) provided by Qiagen (Qiagen, Hilden, Germany) including 5 CpG sites in the promoter region of *IRS1* (Fig. 1). Due to technical reasons (optimization of dispensation order in the PyroMark run) only 4 out of 5 CpG sites were analysed. Pyrosequencing was run on a PyroMark Q24 with subsequent analysis of the obtained results via PyroMark Q24 software, version 2.0.6 (Qiagen, Hilden, Germany). For replication purposes each DNA sample was PCR amplified and analysed twice on different plates. Calculating the interday coefficients of variance (CVs) for replicates results in 0.355 for SAT and 0.298 for OVAT. For statistical analysis the mean percentage of replicates for methylation at every CpG site was determined as well as the mean methylation level across all four analysed CpG sites. Two none template controls per plate containing water were included in PCR reactions and subsequent pyrosequencing analysis.

### Genotyping of rs2943650 near *IRS1*

Genotyping of rs2943650 near the *IRS1* locus was performed in the same 146 individuals for whom methylation analyses were applied (Fig. 1). Genomic DNA was extracted from adipose tissue using GenEluteTM Mammalian Genomic DNA Miniprep Kit (SIGMA-ALDRICH, USA) according to manufacturer,s instructions. Genotypes of rs2943650 were generated by using the KASPar genotyping system (KBioscience allele specific PCR Genotyping System; KBioscience, Teddington, Middlesex, UK). Fluorescence measurement was done with an ABI 7500 Real-Time PCR system. All genotypes were in Hardy-Weinberg equilibrium (*P* > 0.05). The minor allele frequency (MAF) of rs2943650 is 0.42. As we analysed a single SNP missingness per individual is negligible. Errors of genotyping were excluded by random re-genotyping (~5%) of the samples; all genotypes matched the initially designated genotypes. Genotypes of four subjects could not be examined due to technical reasons (uncertainty in allelic discrimination due to low DNA content), which results in a SNP call rate of 97.3%. Water was used as a no template control (NTC).

### Gene expression analysis

mRNA expression data (*IRS1* transcript: ILMN_1874569) was available for 63 subjects (total N = 63, SAT = 56, OVAT = 48) and was extracted from genome wide data achieved by Illumina human HT-12 expression arrays^14^. Corresponding methylation data were generated by pyrosequencing for a subset of these subjects (total N = 60, SAT = 40, OVAT = 32). Paired SAT and OVAT expression data per sample were available for 41 subjects. Complete methylation and expression data in both adipose tissue depots per sample were available for 28 subjects.

### Statistical analysis

We used non-parametric tests as well as bivariate correlation analysis using residuals to adjust for potential confounders such as age, sex and BMI. DNA methylation levels at four individual CpG sites and mean methylation across the entire CpG locus were used as continuous variables. To analyse for differences in methylation/expression levels between OVAT and SAT in the entire cohort and also gender specific, Wilcoxon rank-sum tests were used. Mann-Whitney-U tests were performed to test for differences between obesity subgroups (lean, overweight and obese subgroup). Spearman correlation coefficients were used to assess bivariate correlation (ß = correlation coefficient) with measurements for anthropometric and metabolic traits using residuals to control for covariates sex, age and BMI. Genetic association of rs2943650 with DNA methylation, mRNA expression as well as metabolic and anthropometric traits was tested by using additive (MM vs Mm vs mm; M = major allele, m = minor allele), dominant (MM + Mm vs mm) and recessive modes (MM vs Mm + mm) of inheritance during linear regression analysis adjusted for study specific covariates (sex, age, BMI). All non-normally distributed variables have been log transformed prior to linear regression analysis. Bonferroni correction was used to take into account multiple testing (number of clinical variables*2 adipose tissue depots*3 inheritance models). We lowered the significance threshold to *P* = 4.9 × 10^−4^ (0.05/(17 * 2 * 3) = 4.9 × 10^−4^). Moreover, all *P*-values > 4.9 × 10^−4^ but ≤ 0.05 were considered nominally significant. All *P*-values are provided uncorrected for multiple testing. Statistical analysis were performed using SPSS statistics version 20.0.1 (SPSS, Inc.; Chicago, IL).

### Ethics approval and consent to participate

The study was approved by the Ethics Committee of the University of Leipzig, and performed in accordance to the declaration of Helsinki. All subjects gave written informed consent before taking part in this study.

### Availability of data and material

All data generated or analyzed during this study are included in this published article.

## Results

### rs2943650 near *IRS1* and relationship to clinical variables, *IRS1* mRNA expression and DNA methylation

To dissect whether the reported *IRS1* genetic variant relates to clinically important variables in our cohort we applied genetic association analysis in the total cohort (N = 142; 146 minus four drop outs). Although non-significant, we observed among T allele carriers an unfavourable metabolic profile (such as increased triglyceride levels, increased plasma glucose and insulin levels) along with lower body weight, BMI, waist, hip and visceral and subcutaneous fat area (Table 2) which is in line with previously reported data^1^. We also tested for association between rs2943650 and methylation/expression levels from SAT and OVAT. We observed significantly increased DNA methylation in OVAT among T-allele carriers which withstands adjustment for the covariates sex, age, BMI (Table 2) and also T2D, while *IRS1* mRNA expression is, albeit non-significant, decreased in the same individuals.

**Table 2:** Association between *IRS1* variant rs2943650 and *IRS1* methylation- and expression levels as well as quantitative traits.

	*IRS1* rs2943650		
Genotype (*N*)	TT (49)	TC (67)	CC (26)
**Association analysis with methylation level**			
Mean_4CpGs_SAT	3.23 ± 1.72	3.07 ± 1.54	2.78 ± 1.43
*P*-value	°0.278 #0.249 *0.512		
Mean_4CpGs_OVAT	4.31 ± 2.22	4.26 ± 1.95	3.05 ± 1.4
*P*-value	°0.096 **#0.019** *0.592		
**Association analysis with expression level**			
mRNA expression_SAT	−0.062 ± 0.139	0.057 ± 0.181	0.12 ± 0.126
*P*-value	°0.270 #0.489 *0.303		
mRNA expression OVAT	−0.85 ± 0.123	0.003 ± 0.11	0.023 ± 0.229
*P*-value		°0.101 #0.323 *0.144	
**Association analysis with anthropometric traits**			
Weight (kg)	97.9 ± 43.6	100.4 ± 43.5	111.5 ± 42.9
*P*-value		°0.893 #0.398 *0.621	
Visceral fat area (cm²)	163 ± 142	182 ± 176	215 ± 152
*P*-value	°0.840 #0.298 *0.557		
Subcutaneous fat area (cm²)	564 ± 687	751 ± 801	991 ± 829
*P*-value	°0.482 #0.295 *0.868		
CT-ratio (OVAT/SAT fat area)	0.96 ± 1.36	0.52 ± 0.51	0.47 ± 0.51
*P*-value	°0.669 #0.942 *0.559		
BMI (kg/m²)	34 ± 15	36 ± 15	38 ± 15
*P*-value	°0.459 #0.564 *0.534		
Waist circumference (cm)	104.8 ± 29.5	104.6 ± 33.8	118.3 ± 30.2
*P*-value	°0.996 #0.128 *0.197		
Hip circumference (cm)	109.2 ± 25.7	113.0 ± 31.2	124.9 ± 33.6
*P*-value	°0.620 #0.446 *0.165		
Waist-to-hip ratio	0.95 ± 0.13	0.92 ± 0.12	0.95 ± 0.11
*P*-value	°0.707 #0.342 *0.813		
body fat (%)	30.2 ± 13.5	32.3 ± 13.2	29.9 ± 9.1
*P*-value	°0.445 #0.346 *0.675		
**Association analysis with glucose/insulin metabolism**			
Fasting plasma glucose (mmol/l)	6.41 ± 1.77	5.84 ± 1.93	5.85 ± 1.33
*P*-value	°0.457 #0.813 *0.195		
2 h OGTT plasma glucose (mmol/l)	6.80 ± 2.17	7.18 ± 3.47	6.56 ± 1.43
*P*-value	°0.975 #0.714 *0.798		
HbA1c (%)	5.98 ± 0.92	5.68 ± 0.64	5.83 ± 0.77
*P*-value	°0.824 #0.406 *0.319		
Fasting plasma Insulin (pmol/l)	97.74 ± 164.59	62.45 ± 105.66	70.46 ± 68.3
*P*-value	°0.988 #0.119 *0.166		
**Association analysis with lipid metabolism**			
HDL cholesterol (mmol/l)	1.33 ± 0.46	1.4 ± 0.47	1.31 ± 0.31
*P*-value	°0.846 #0.810 *0.923		
LDL cholesterol (mmol/l)	3.39 ± 1.04	3.19 ± 1.17	3.13 ± 0.85
*P*-value	°0.694 #0.955 *0.532		
Triglycerides (mmol/l)	1.49 ± 0.72	1.35 ± 0.85	1.30 ± 0.47
*P*-value	°0.659 #0.961 *0.544		
Adiponectin (µg/ml)	7.96 ± 4.54	10.17 ± 6.18	8.39 ± 5.17
*P*-value		°0.541 #0.672 *0.229	

N = number f subjects (total cohort N = 142) (T2D: N = 42 with TT = 19; TC = 16; CC = 7); *P*-values were calculated using °additive (MM vs Mm vs mm), ^#^dominant (MM + Mm vs mm) and *recessive (MM vs Mm + mm) modes of inheritance by linear regression analysis adjusted for age, sex and logBMI (except for BMI). Nominal significant *P*-values are highlighted in bold. SAT = subcutaneous adipose tissue, OVAT = omental visceral adipose tissue; BMI = body mass index; OGTT = oral glucose tolerance test; HDL = high density lipoprotein cholesterol; LDL = low density lipoprotein cholesterol.

### Inter-depot specific DNA methylation within *IRS1* promoter region

**Figure 2:**
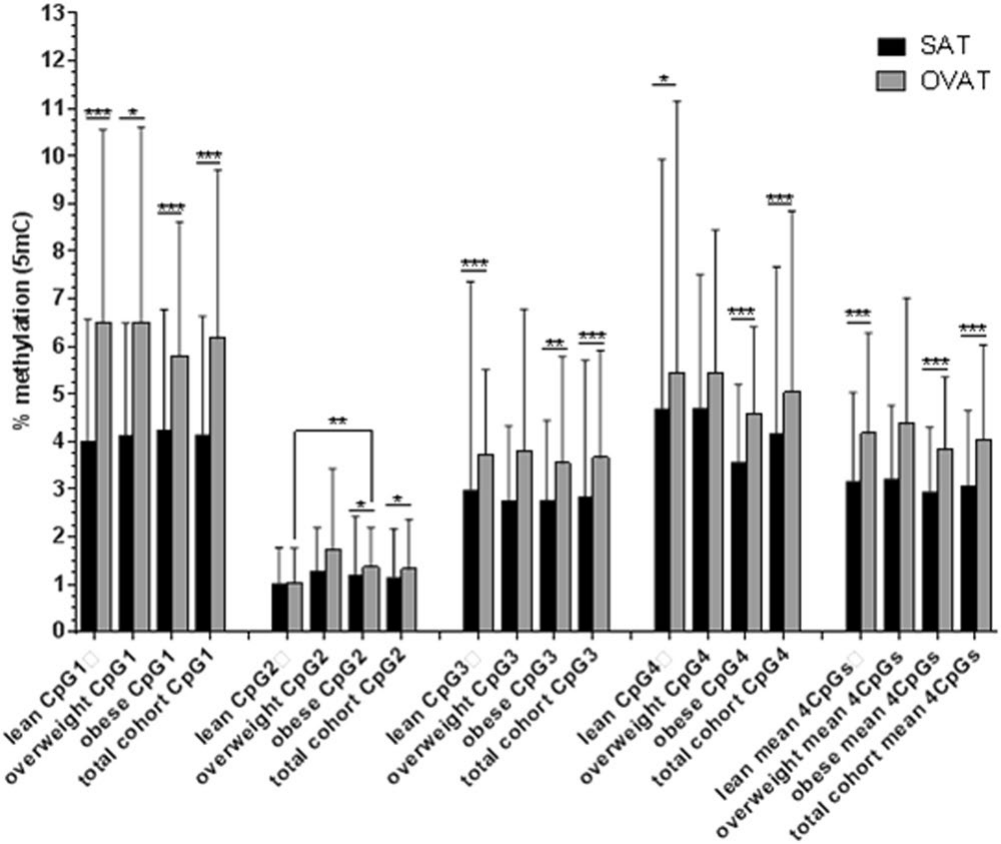
DNA methylation at one CpG locus within *IRS1* promoter in SAT vs OVAT in BMI subgroups. Data are presented as mean ± standard deviation (S.D.). Number of participants: lean = 50; overweight = 28; obese = 68; total cohort = 146. *P*-values were calculated using non-parametric tests; significance of *p*-values are indicated as following *<0.05; **<0.001; ***<0.0001. 5mC = 5methyl cytosine; SAT = subcutaneous adipose tissue; OVAT = omental visceral adipose tissue.

We measured *IRS1* DNA methylation at one CpG-locus including 4 CpG sites within the promoter region of *IRS1* in 146 paired samples of SAT and OVAT. In the entire cohort, mean SAT methylation (average of all 4CpGs = 3.08 ± 1.57%) was significantly lower than OVAT methylation (average of all 4CpGs = 4.05 ± 1.98%, *P* < 0.0001, Fig. 2) which was also nominally significant for all 4 CpG sites separately (all *P* < 0.05). Similar directions were observed in BMI stratified subgroups of lean, overweight and obese individuals. CpG2 in OVAT is the only site representing slightly significant higher methylation levels in obese than in lean subjects (*P* = 0.003). Results are shown in Fig. 2.

Furthermore, no significant methylation differences were observed between subjects with (N = 42) and without type 2 diabetes (N = 104; data not shown). In the entire cohort (N = 146) the described inter-depot specific differences in *IRS1* DNA methylation levels between SAT and OVAT are stronger in women (men *P* = 0.001; women *P* = 3.8 × 10^−5^, data not shown) which might be due to differences in sample sizes (men N = 55; women N = 91).

### DNA methylation correlates with measures of fat distribution and glucose/lipid metabolism

To assess potential relationships of *IRS1* promoter methylation and metabolic and anthropometric variables, we performed bivariate Spearman correlation analysis in all samples (N = 146). By analysing BMI stratified subgroups we observed in obese individuals that OVAT methylation positively correlates with variables of fat distribution such as waist (*P* = 0.006; ß = 0.349), and WHR (*P* = 0.038; ß = 0.266) and markers of glucose metabolism such as fasting plasma glucose (*P* = 0.026; ß = 0.271). HDL-cholesterol levels are negatively related to OVAT methylation (*P* = 0.013; ß = −0.415).

Further, in lean subjects, SAT methylation positively correlates with HDL cholesterol (*P* = 0.031; ß = 0.341) while OVAT methylation is related negatively to body fat percentage (P = 0.032; ß = −0.373).

In the overweight subgroup, HDL-cholesterol is negatively related to SAT methylation (*P* = 0.044; ß = −0.444) and subcutaneous fat area is negatively correlated with OVAT methylation (*P* = 0.041; ß = −0.412). All data are adjusted for sex, age and BMI and are summarized in Table 3. Additional adjustment for T2D results in the loss of association to FPG (data not shown). However, none of the here reported results withstands correction for multiple testing.

**Table 3:** Correlation analysis of mean CpG locus methylation with quantitative phenotypes.

Quantitative trait	N*	Total cohort ( *N* = 146)		Lean (N = 50)		Overweight (N = 28)		Obese (N = 68)	
		Mean CpG locus methylation in							
		SAT	OVAT	SAT	OVAT	SAT	OVAT	SAT	OVAT
Weight	146/50/28/68	0.813^a^ [0.020]^b^	0.467 [−0.061]	0.061 [−0.267]	0.846 [0.028]	0.175 [0.264]	0.130 [−0.293]	0.490 [0.085]	0.717 [0.045]
Visceral fat area (cm²)	135/49/25/61	0.928 [−0.008]	0.716 [0.032]	0.502[0098]	0.435 [0.114]	0.060 [−0.382]	0.216 [−0.256]	0.871 [0.021]	0.222 [0.159]
Subcutanoues fat area (cm²)	135/49/25/61	0.181 [0.116]	0.948 [−0.006]	0.134 [0.217]	0.862 [−0.026]	0.875 [−0.033]	**0.041 [**−**0.412]**	0.411 [0.107]	0.665 [0.057]
CT−ratio (OVAT/SAT fat area)	135/49/25/61	0.633 [0.042]	0.272 [0.095]	0.959 [0.008]	0.723 [−0.052]	0.852 [0.039]	0.230 [0.249]	0.247 [0.151]	0.595 [−0.069]
BMI (kg/m^2^)	146/50/28/68	0.469 [0.060]	0.731 [0.029]	0.790 [−0.039]	0.145 [−0.209]	0.301 [0.203]	0.312 [0.198]	0.318 [0.123]	0.210 [−0.154]
Waist circumference (cm)	139/50/28/61	0.372 [0.076]	0.280 [0.092]	0.736 [0.049]	0.536 [−0.090]	0.595 [0.105]	0.327 [−0.192]	0.636 [0.062]	**0.006 [0.349]**
Hip circumference (cm)	139/50/28/61	0.088 [0.145]	0.603 [−0.044]	0.512 [0.095]	0.259 [−0.163]	0.353 [0.182]	0.816 [−0.046]	0.294 [0.136]	0.847 [−0.025]
Waist-to-hip ratio	139/50/28/61	0.873 [0.014]	0.574 [0.048]	0.878 [0.022]	0.467 [−0.105]	0.857 [−0.036]	0.446 [−0.150]	0.863 [−0.023]	**0.038 [0.266]**
Body fat (%)	81/33/15/33	0.161 [−0.157]	0.384 [−0.098]	0.370 [0.161]	**0.032 [**−**0.373]**	0.071 [−0.479]	0.830 [−0.061]	0.074 [−0.315]	0.875 [0.028]
Fasting plasma glucose (mmol/l)	146/50/28/68	0.590 [0.045]	0.168 [0.115]	0.419 [0.117]	0.671 [−0.062]	0.551 [0.118]	0.914 [0.021]	0.943 [−0.009]	**0.026 [0.271]**
2 hr OGTT plasma glucose (mmol/l)	75/32/10/33	0.510 [0.077]	0.980 [−0.003]	0.465 [0.134]	0.595 [−0.098]	0.533 [−0.224]	0.777 [−0.103]	0.519 [0.116]	0.856 [0.033]
Fasting plasma Insulin (pmol/l)	123/46/25/52	0.838 [0.019]	0.757 [0.028]	0.371 [0.135]	0.595 [−0.097]	0.606 [−0.108]	0.679 [−0.087]	0.981 [−0.003]	0.179 [0.189]
HbA1c (%)	137/49/27/61	0.798 [0.022]	0.775 [0.025]	0.386 [0.127]	0.774 [−0.042]	0.626 [0.098]	0.417 [−0.163]	0.585 [−0.071]	0.114 [0.204]
HDL cholesterol (mmol/l)	96/40/21/35	0.697 [0.040]	0.083 [−0.178]	**0.031 [0.341]**	0.631 [−0.078]	**0.044 [**−**0.444]**	0.695 [0.091]	0.799 [−0.045]	**0.013 [−0.415]**
LDL cholesterol (mmol/l)	93/41/21/31	0.931 [0.009]	0.310 [−0.106]	0.241 [0.187]	0.121 [−0.246]	0.427 [−0.183]	0.729 [−0.081]	0.660 [−0.082]	0.236 [0.219]
Triglycerides (mmol/l)	86/32/18/36	0.806 [0.027]	0.696 [−0.043]	0.973 [0.006]	0.130 [−0.273]	0.179 [0.331]	0.345 [−0.236]	0.621 [−0.085]	0.293 [0.180]
Adiponectin (µg/ml)	98/32/17/49	0.777 [−0.029]	0.642 [0.048]	0.068 [−0.326]	0.938 [−0.014]	0.256 [0.292]	0.765 [0.078]	0.729 [0.051]	0.429 [0.116]

WHO classification: lean ≥ 18; <25 kg/m²; overweight ≥ 25 kg/m²; <30 kg/m²; obese ≥ 30 kg/m²; SAT (subcutaneous adipose tissue); OVAT (omental visceral adipose tissue), N = subjects (N* in total cohort/lean/overweight/obese individuals, respectively); a = P-value. P-values were calculated using bivariate Spearman correlation analysis (adjusted for age, sex and BMI (except for BMI) by calculating standardized residuals); nominal significant P-values are highlighted in bold. b = beta (ß; effect size and direction), BMI = body mass index; OGTT = oral glucose tolerance test; HDL = high density lipoprotein cholesterol; LDL = low density lipoprotein cholesterol.

### *IRS1* mRNA expression is adipose tissue depot specific

**Figure 3:**
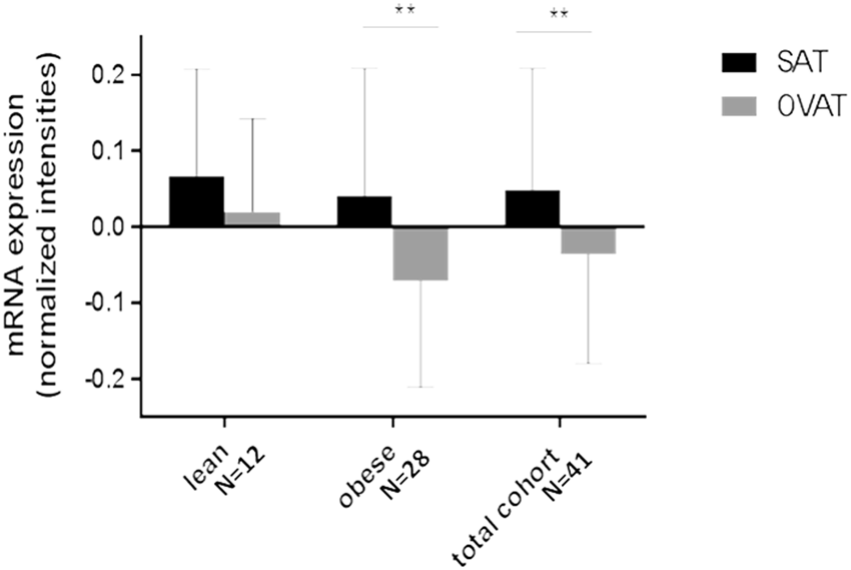
*IRS1* mRNA expression in SAT and OVAT. Total N = 41 represent paired SAT and OVAT expression data per sample. Overweight subgroup was excluded due to small sample size (N = 1). Data are presented as mean ± standard deviation. P-values were calculated using non-parametric t-tests. SAT = subcutaneous adipose tissue; OVAT = omental visceral adipose tissue.

Expression data either from SAT or OVAT were extracted from genome wide expression data reported previously^14^ for 63 subjects. To compare SAT and OVAT expression level, we lowered the sample size to N = 41 – subjects representing paired SAT and OVAT expression data. We observed significantly higher *IRS1* mRNA expression in SAT compared to OVAT in the entire cohort (N = 41; *P* = 0.0004) and obese individuals (N = 28; *P* = 0.001; Fig. 3) while this relationship is non-significant in lean subjects. No significant differences in *IRS1* mRNA expression were detected in men vs women while T2D patients (N = 10) show lower *IRS1* expression ( = mean −0.04 ± SD 0.14) than subjects without T2D (N = 31; = mean 0.08 ± SD 0.15) in SAT (*P* = 0.032).

### *IRS1* mRNA expression and DNA methylation are correlated in obese subjects

**Figure 4:**
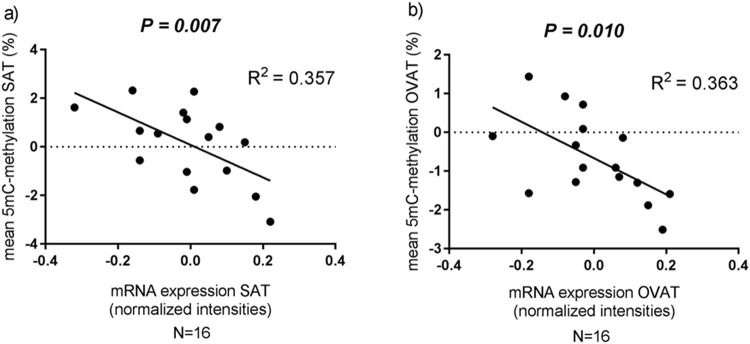
Correlation analysis of *IRS1* DNA methylation and mRNA gene expression in SAT and OVAT among obese subjects. (**a**) correlation analysis in SAT; (**b**) correlation analysis in OVAT. Data were calculated by using Spearman correlation analysis by using standardized residuals to adjust for age, sex and BMI. 5mC = 5methyl cytosine; SAT = subcutaneous adipose tissue; OVAT = omental visceral adipose tissue.

In order to better understand whether DNA methylation may impact on gene expression we performed correlation analysis in all individuals for whom DNA methylation and expression were available in both SAT and OVAT (*N* = 28). Among obese individuals (*N* = 16), DNA methylation of *IRS1* and its gene expression are negatively correlated in both SAT and OVAT (Fig. 4) and withstand adjustment for T2D (data not shown).

### *IRS1* mRNA expression is related to variables of glucose metabolism

To analyse whether the *IRS1* mRNA expression relates to clinically relevant variables, we conducted bivariate Spearman correlation analysis in the total cohort and in BMI stratified subgroups. In order to compare results, these analysis were performed among all 60 individuals from whom overlapping methylation and expression data in either SAT or OVAT were available. All data were adjusted for age, sex and BMI (summarized in Table 3). Albeit not statistically significant we observed in the entire cohort and in subgroups nominal significant relationships of *IRS1* expression levels and variables of glucose metabolism such as fasting and/or 2 h plasma glucose levels (total cohort: fasting plasma glucose (*P* = 0.046; ß = −0.317); lean: fasting plasma glucose (*P* = 0.046; ß = −0.463) and 2 h plasma glucose levels (*P* = 0.023; ß = −0.582); obese: 2 h plasma glucose levels (*P* = 0.047; ß = 0.714). Only the association between SAT *IRS1* mRNA expression and FPG in lean individuals survives additional adjusting for T2D (*P* = 0.021; ß = −0.589, data not shown). However, none of these associations would withstand correction for multiple testing. Moreover, they confer different effect directions between obese individuals and the remainder (Table 4

**Table 4:** Correlation analysis between *IRS1* mRNA expression and quantitative phenotypes.

Quantitative trait	N*	**Total cohort (N = 60)**		**Lean (N = 23)**		**Obese (N = 34)**	
		***mean IRS1*** **mRNA expression in**					
		SAT	OVAT	SAT	OVAT	SAT	OVAT
	*SAT/OVAT*						
	*total – lean – obese*						
Weight	40/32 – 19/14 – 19/17	0.270^a^ [0.179]^b^	0.239 [−0.214]	0.663 [0.107]	0.464 [−0.213]	0.473 [0.175]	0.451 [−0.196]
Visceral fat area (cm²)	34/27 – 19/14 – 13/12	0.260 [−0.198]	0.058 [0.370]	0.229 [−0.289]	0.474 [0.209]	0.201 [−0.379]	0.255 [0.357]
Subcutanoues fat area (cm²)	34/27 – 19/14 – 13/12	0.890 [0.025]	0.249 [−0.230]	0.842 [0.049]	0.899 [−0.037]	0.494 [0.209]	0.208 [−0.392]
CT-ratio (OVAT/SAT fat area)	34/27 – 19/14 – 13/12	0.713 [−0.066]	0.866 [0.034]	0.943 [0.018]	0.455 [0.218]	0.803 [0.077]	0.779 [0.091]
BMI kg/m^2^	40/32 – 19/14 – 19/17	0.274 [−0.177]	0.236 [−0.216]	0.065 [0.432]	0.605 [0.152]	0.344 [−0.230]	0.248 [−0.297]
Waist circumference (cm)	34/27 – 19/14 – 13/12	0.701 [−0.068]	0.696 [−0.079]	0.391 [−0.209]	0.681 [−0.121]	0.873 [0.049]	0.795 [−0.084]
Hip circumference (cm)	34/27 – 19/14 – 13/12	0.926 [0.017]	0.583 [−0.111]	0.586 [0.133]	0.543 [−0.178]	0.845 [0.060]	0.430 [−0.252]
Waist-to-hip ratio	34/27 – 19/14 – 13/12	0.598 [−0.094]	0.854 [−0.037]	0.057 [−0.444]	0.899 [0.037]	0.721 [0.110]	0.914 [−0.035]
Body fat (%)	23/18 – 11/8 – 11/10	0.511 [−0.144]	0.829 [−0.055]	0.484 [0.236]	0.823 [−0.095]	0.650 [0.155]	0.881 [0.055]
Fasting plasma glucose (mmol/l)	40/32 – 19/14 – 19/17	**0.046** [−**0.317]**	0.655[−0.082]	**0.046** [−**0.463]**	0.840 [−0.059]	0.088 [−0.402]	0.866 [−0.044]
2 hr OGTT plasma glucose (mmol/l)	25/17 – 15/8 – 8/8	0.303 [−0.215]	0.135 [0.377]	**0.023** [−**0.582]**	0.911 [0.048]	0.352 [0.381]	**0.047 [0.714]**
Fasting plasma Insulin (pmol/l)	38/29 – 19/13 – 17/15	0.282 [−0.179]	0.190 [0.251]	0.367 [−0.219]	0.972 [0.011]	0.593 [−0.140]	0.405 [0.232]
HbA1c (%)	39/31 – 19/14 – 18/16	0.382 [−0.144]	0.763 [0.056]	0.178 [−0.323]	0.615 [0.147]	0.576 [−0.141]	0.854 [0.050]
HDL cholesterol (mmol/l)	27/22 – 15/11 – 10/10	0.765 [0.060]	0.334 [−0.216]	0.820 [0.064]	0.160 [−0.455]	0.651 [−0.164]	0.934 [−0.030]
LDL cholesterol (mmol/l)	27/23 – 15/12 – 10/10	0.947 [0.013]	0.477 [0.156]	1.000 [0.000]	0.245 [0.364]	0.726 [−0.127]	0.446 [0.273]
Triglycerides (mmol/l)	22/18 – 11/8 – 10/9	0.393 [−0.191]	0.705 [0.096]	0.937 [0.027]	0.823 [−0.095]	0.934 [0.030]	0.668 [0.167]
Adiponectin (µg/ml)	26/21 – 12/9 – 13/12	0.350 [0.191]	0.475 [−0.165]	0.880 [−0.049]	0.460 [−0.283]	0.061 [0.533]	0.319 [−0.315]

all subjects representing methylation and expression data in either SAT (N = 40) or OVAT (N = 32) have been included while N* = subjects in SAT/OVAT per group (total – lean *–*obese); overweight subgroup was excluded due to N = 3; WHO classification: lean≥18; <25 kg/m²; <30 kg/m²obese≥ 30 kg/m²; SAT (subcutaneous adipose tissue); OVAT (omental visceral adipose tissue), a = P-value. P-values were calculated using bivariate Spearman correlation analysis (adjusted for age, sex and BMI (except for BMI) by calculating standardized residuals); nominal significant P-values are highlighted in bold. b = beta (ß; effect size and direction) BMI = body mass index; OGTT = oral glucose tolerance test; HDL = high density lipoprotein cholesterol; LDL = low density lipoprotein cholesterol.

).

## Discussion

Body fat distribution is strongly related to obesity associated co-morbidities^4^. While large GWAS studies have identified numerous SNPs associated with WHR and body fat percentage, it is poorly understood how these variants influence metabolic traits^2,23^. Interestingly, many GWAS variants were identified as being methylation quantitative trait loci (meQTL) indicating that the interplay between genetics and epigenetics is important for disease development^24^. Moreover, nearly half of the identified T2D risk variants, including one variant of *IRS1* (rs7578326), are CpG-SNPs with an influence on methylation status of these SNP sites and/or surrounding CpG sites, hence might have an impact on gene expression^16^.

Here, we tested whether genotypes at a previously identified body fat associated SNP variant near *IRS1* rs2943650 interplay with *IRS1* DNA methylation and gene expression, thereby potentially mediating the observed effects on individual metabolic profiles and body fat distribution. We genotyped rs2943650 and measured DNA methylation in 146 subjects. Additionally, *IRS1* mRNA expression data were extracted from a previous data set^14^. Although non-significant, which is most likely due to the small sample size for genetic association studies, our data are in line with others^1,25^. We found among T allele carriers (major allele) a favourable anthropometric pattern, which is however combined with more unhealthy metabolic variables compared to C allele carriers. These results largely support the previously reported hypothesis that a relative decrease in subcutaneous to visceral adipose tissue mass is related to e.g. insulin resistance, dyslipidemia and lower adiponectin levels^1^. Our data further suggest that *IRS1* DNA methylation at several CpG sites within the promoter is strongly fat depot specific with significantly higher levels in OVAT. Interestingly, we found that rs2943650 T-allele is related to increased methylation in OVAT while OVAT DNA methylation levels correlate with variables of fat distribution and glucose/lipid metabolism. Interestingly, this relationship may be obesity specific as we observed opposite effect directions of OVAT DNA methylation on e.g. body fat in obese compared to lean individuals; while in general obesity-specific methylation effects were reported in many studies^26^. In the light of these results one could speculate, that the observed effects on fat distribution are, at least partially and tissue specifically, driven by a potential interplay between genetic variation and *IRS1* DNA methylation.

Future studies could focus on the use of both, a patient’s SNP and DNA methylation data as diagnostic tool for the development of adiposity related co-morbidities like T2D. Hence, since OVAT isn’t easy accessible it would be interesting to know whether DNA methylation level of *IRS1* in blood reflect those derived in OVAT. However, larger studies are warranted which address these questions but also include further SNP markers, their potential role in predicting DNA methylation and the use as diagnostic tool.

Since both, DNA methylation and SNP variants can influence gene expression we tested for associations with *IRS1* mRNA expression. *IRS1* mRNA is differentially expressed in SAT and OVAT with lower levels in visceral adipose tissue depot while T-allele carriers show lower *IRS1* mRNA expression in OVAT. Further, among obese individuals *IRS1* DNA methylation is negatively related to *IRS1* gene expression in SAT and OVAT. We therefore conclude that both SNP and *IRS1* DNA methylation are involved in regulating depot-specific *IRS1* mRNA expression which might be more prominent among obese subjects. Taken together, our data suggest that the mechanisms underlying the association between rs2943650 near *IRS1* and body composition measures may be mediated by promoter hyper-methylation among risk allele carriers in a tissue specific manner. Further, DNA methylation and gene expression are most strongly correlated among obese individuals underlining that this relationship may be obesity-specific. Possible mechanisms underlying the observed results may also include microRNA expression which was previously described to be adipose tissue depot-specific^27–29^. This can result in depot-specific *IRS1* mRNA translation in SAT and OVAT which consequently affects protein content and subsequently metabolic traits tissue specifically.

Our study has several limitations including the small sample size. We included 146 individuals in the genetic association study for whom we measured DNA methylation data. Although the number of individuals is sufficient for studying epigenetic effects it is too small to identify genetic associations. This is most likely the reason for the lack of statistically significant results in SNP analysis, although they show the same trend and effect directions as larger previously published studies. In addition, expression data were only available for a subgroup of subjects which can lead to false positive or false negative results. We only measured DNA methylation at 4 CpG sites which may not be representative for the entire promoter region. Moreover, we used adipose tissue biopsies which naturally contain several cell types. Therefore, it cannot be ruled out that effects from other cell types such as macrophages may have influenced our results. Finally, we do not have information about nutrition, smoking and other environmental factors that may impact on epigenetic mechanisms. However, T2D and medication do not affect the shown associations.

In conclusion, our results suggest, that the previously reported body fat associated variant rs2943650 (T) interacts with DNA hypermethylation in OVAT at several CpG sites within the *IRS1* promoter linking epigenetic and genetic effects. Since DNA methylation and gene expression are negatively correlated in both SAT and OVAT among obese individuals, the observed findings might be obesity-specific. However, the interplay of genetic and epigenetic factors at the *IRS1* locus seems do not sufficiently explain the overall variability of metabolic alterations which clearly indicates that other mechanisms need to be taken into account.

### Perspectives

- Individual adipose tissue distribution is a critical parameter for developing obesity-related co-morbidities such as type 2 diabetes mellitus. Many studies identified genetic risk variants related to fat distribution. However, underlying mechanisms on how these variants influence the risk for co-morbidities still needs to be explored and will add substantial clinical relevance for the treatment of patients.
- This study identifies *IRS1* to be differentially methylated and expressed in human SAT and OVAT which correlates to metabolic and anthropometric variables. Furthermore, higher OVAT methylation in obese individuals associates with the previously identified risk variant for fat distribution rs2943650 providing insights into genetic and epigenetic interactions on human fat distribution.
- IRS1 is a major player in insulin signalling. Hence, understanding on how genetic variants near or within its gene influence the metabolism alone or in concert with epigenetic alterations will help us identifying clinically relevant underlying mechanisms related to metabolic co-morbidities.
